# Supplementary material for: Evolution and spatial reconstruction of rural settlements based on composite features of agglomeration effect and ecological effects in the Hexi Corridor, Northwest China
Source: PLoS One. 2023 Nov 8;18(11):e0294037. doi: 10.1371/journal.pone.0294037 (PMC10631663; doi:10.1371/journal.pone.0294037)
Supplement: Attachment — Submitted filename: Reviewers.docx [file pone.0294037.s001.docx]

**Manuscript #: PONE-D-23-19951**

**Title:** Evolution and Spatial Reconstruction of Rural Settlements with Combined Agglomeration and Ecological Value Characteristics in the Hexi Corridor, Northwest China

**Authors：**Xiaoying nie; Chao wang; Wanzhuang huang

**Article type:** Research Article

**Reviewer:**

In this manuscript, the author applied GIS spatial analysis technology and ecosystem service value modeling to analyze the combined spatial agglomeration and ecological value characteristics of rural settlements in the Hexi Corridor, Northwest China. The study then identifies specific rural settlement reconstruction zoning and optimization directions based on rural settlement accessibility. This study is interesting and provides a related dataset. However, there are some areas that need improvement, which are listed below.

**Major:**
1. Line 425-427: Is it feasible to determine the boundary of the reconfiguration range of rural settlements based on the accessibility measurement method, considering the spatial distribution and size of the reconstituted zones?
2. Line 530: Please explain the meaning of the statistic “85.52%” and provide clarification on its significance.

**Minor:**
1. Line 108-109: All numbers above 1000 should include thousands separators, such as “1,000”.
2. Line 123: Replace “Gansu Province” with “Hexi Corridor”.
3. Line 145: Add “in China” after “Statistics”.
4. Line 156: The “Ecosystem services value equivalent factor” column in Table 2 should be placed in a separate table between line 219 and line 220.

5. Line 160 and Line 258: Replace “NNI” with the correct term.
6. Line 262: Consider separating the paragraph starting with “The trend surface analysis…” into separate paragraphs.
7. Line 291-298: Merge the paragraph starting with “We detected two density…” with the previous paragraph.
8. Line 313-322: Merge the paragraph starting with “Rural settlement cold spots…” with the previous paragraph.
9. Line 349: There is an error in the format “3-km × 3-km”. Please correct it.
10. Line 392: The format “0.68·km-2 to 1.41·km-2” is incorrect. Please revise it.
11. Line 439: Replace “(” with “,”.
12. Line 454- 460: Consider separating the segments of “Central village building…” to “efficient agriculture” into separate paragraphs.
13. Line 461-470: Consider separating the segments of “The internally coordinated…” to “residents” into separate paragraphs.
14. Line 470-481: Consider separating the segments of “With the purpose…” to “rural revitalization and development” into separate paragraphs.
15. Line 538: Replace “.” with “;” to improve clarity.
