# Supplementary material for: Evolution and spatial reconstruction of rural settlements based on composite features of agglomeration effect and ecological effects in the Hexi Corridor, Northwest China
Source: PLoS One. 2023 Nov 8;18(11):e0294037. doi: 10.1371/journal.pone.0294037 (PMC10631663; doi:10.1371/journal.pone.0294037)
Supplement: Attachment — Submitted filename: Response to Reviewers.doc [file pone.0294037.s002.doc]

**PONE-D-23-19951**

**Evolution and Spatial Reconstruction of Rural Settlements with Combined Agglomeration and Ecological Value Characteristics in the Hexi Corridor, Northwest China**

**PLOS ONE**

**To Editor:**

Thank you for giving us a chance to revise the manuscript. We also thank the reviewers for their constructive suggestions to help us improve the quality of the manuscript. We have made changes in the manuscript according to the reviewers’ comments and your advice. The revised manuscript has utilized “tracked changes” to identify the revisions. A clean manuscript is also provided. We hope these changes make the manuscript acceptable for publication.

1. Please ensure that your manuscript meets PLOS ONE's style requirements, including those for file naming. The PLOS ONE style templates can be found at

<https://journals.plos.org/plosone/s/file?id=wjVg/PLOSOne_formatting_sample_main_body.pdf> and

<https://journals.plos.org/plosone/s/file?id=ba62/PLOSOne_formatting_sample_title_authors_affiliations.pdf>

Response:

Thanks for your comments. It has been modified according to Plos One journal template requirements.

2. In your Methods section, please provide additional information regarding the permits you obtained for the work. Please ensure you have included the full name of the authority that approved the field site access and, if no permits were required, a brief statement explaining why.

Response:

Thanks for your comments. All the methods in this paper need ArcGIS10.5 software to complete, and we have t permits to use the software.

3. Thank you for stating the following financial disclosure:

“This research was funded by the Science and Technology Program of Gansu Province (grant number 20JR10RA296).”

Please state what role the funders took in the study. If the funders had no role, please state: "The funders had no role in study design, data collection and analysis, decision to publish, or preparation of the manuscript."

If this statement is not correct you must amend it as needed.

Please include this amended Role of Funder statement in your cover letter; we will change the online submission form on your behalf.

Response:

Thanks for your comments. Our cover letter include this amended Role of Funder statement.

Grants and funding: This research was funded by the Science and Technology Program of Gansu Province (grant number 20JR10RA296). The funders had no role in study design, data collection and analysis, decision to publish, or preparation of the manuscript.

4. We note that Figures 1, 3, 4, 5 and 6 in your submission contain [map/satellite] images which may be copyrighted. All PLOS content is published under the Creative Commons Attribution License (CC BY 4.0), which means that the manuscript, images, and Supporting Information files will be freely available online, and any third party is permitted to access, download, copy, distribute, and use these materials in any way, even commercially, with proper attribution. For these reasons, we cannot publish previously copyrighted maps or satellite images created using proprietary data, such as Google software (Google Maps, Street View, and Earth). For more information, see our copyright guidelines: http://journals.plos.org/plosone/s/licenses-and-copyright.

We require you to either (1) present written permission from the copyright holder to publish these figures specifically under the CC BY 4.0 license, or (2) remove the figures from your submission:

1. You may seek permission from the original copyright holder of Figures 1, 3, 4, 5 and 6 to publish the content specifically under the CC BY 4.0 license.

We recommend that you contact the original copyright holder with the Content Permission Form (http://journals.plos.org/plosone/s/file?id=7c09/content-permission-form.pdf) and the following text:

“I request permission for the open-access journal PLOS ONE to publish XXX under the Creative Commons Attribution License (CCAL) CC BY 4.0 (http://creativecommons.org/licenses/by/4.0/). Please be aware that this license allows unrestricted use and distribution, even commercially, by third parties. Please reply and provide explicit written permission to publish XXX under a CC BY license and complete the attached form.”

Please upload the completed Content Permission Form or other proof of granted permissions as an "Other" file with your submission.

In the figure caption of the copyrighted figure, please include the following text: “Reprinted from [ref] under a CC BY license, with permission from [name of publisher], original copyright [original copyright year].”

2. If you are unable to obtain permission from the original copyright holder to publish these figures under the CC BY 4.0 license or if the copyright holder’s requirements are incompatible with the CC BY 4.0 license, please either i) remove the figure or ii) supply a replacement figure that complies with the CC BY 4.0 license. Please check copyright information on all replacement figures and update the figure caption with source information. If applicable, please specify in the figure caption text when a figure is similar but not identical to the original image and is therefore for illustrative purposes only.c

The following resources for replacing copyrighted map figures may be helpful:

USGS National Map Viewer (public domain): http://viewer.nationalmap.gov/viewer/

The Gateway to Astronaut Photography of Earth (public domain): http://eol.jsc.nasa.gov/sseop/clickmap/

Maps at the CIA (public domain): https://www.cia.gov/library/publications/the-world-factbook/index.html and https://www.cia.gov/library/publications/cia-maps-publications/index.html

NASA Earth Observatory (public domain): http://earthobservatory.nasa.gov/

Landsat: http://landsat.visibleearth.nasa.gov/

USGS EROS (Earth Resources Observatory and Science (EROS) Center) (public domain): http://eros.usgs.gov/#

Natural Earth (public domain): <http://www.naturalearthdata.com/>

Response:

Thanks for your comments. The data in figures 1, 3, 4, 5 and 6 of the paper is can be obtained from the Data Center for Resources and Environmental Sciences, Chinese Academy of Sciences (RESDC) ([http://www.resdc.cn](http://www.resdc.cn/)); the Geospatial Data Cloud site, Computer Network Information Center, Chinese Academy of Sciences(http://www.gscloud.cn) and China’s geographic remote sensing ecological network platform ([www.gisrs.cn](http://www.gisrs.cn/)). A data availability statement is provided in S1 File.

5. Please review your reference list to ensure that it is complete and correct. If you have cited papers that have been retracted, please include the rationale for doing so in the manuscript text, or remove these references and replace them with relevant current references. Any changes to the reference list should be mentioned in the rebuttal letter that accompanies your revised manuscript. If you need to cite a retracted article, indicate the article’s retracted status in the References list and also include a citation and full reference for the retraction notice.

Response:

Thanks for your comments. We have carefully checked the references listed in the paper to make sure that it is complete and correct.

**To Reviewer:**

We are very grateful for your assessment of our work. Thank you very much for your time and effort in reviewing our manuscript. We appreciate your useful comments. Please find our responses to the reviewer’s suggestions in the following section.

**Reviewer #1:**

This research is really sound and informative to the international reader. However here is some comment to more improve the paper before published.

1. Improve outline of the paper for more attractive

Response:

Thanks for your comments. We have revised the outline of the paper according to the structure and template requirements of Plos One journal.

2. the paragraph are too long and unattractive for the reader please be condense and make them more readable

Response:

Thanks for your comments. It has been modified according to your suggestion.

3. paper needs future suggestion

Response:

Thanks for your comments. We have added future suggestion after the conclusion of the paper.

4.abstract need rewrite

Response:

Thanks for your comments. It has been modified according to your suggestion.

5. please give care for research methodology and its outline

Response:

Thanks for your comments. It has been modified according to your suggestion.

6.Likely to research methodology, I wanna to be you give strong attention your result part. please make it more informative, readable, clear based on your data

Response:

Thanks for your comments. It has been modified according to your suggestion.

7. Make your title more clear and informative for international knowledge searcher in this field of discipline

Response:

Thanks for your comments. We have modified the title to “Evolution and Spatial Reconstruction of Rural Settlements Based on Composite Features of Agglomeration Effect and Ecological Effects in the Hexi Corridor, Northwest China”.

**Reviewer #2:**

In this manuscript, the author applied GIS spatial analysis technology and ecosystem service value modeling to analyze the combined spatial agglomeration and ecological value characteristics of rural settlements in the Hexi Corridor, Northwest China. The study then identifies specific rural settlement reconstruction zoning and optimization directions based on rural settlement accessibility. This study is interesting and provides a related dataset.

**Major:**

1. Line 425-427: Is it feasible to determine the boundary of the reconfiguration range of rural settlements based on the accessibility measurement method, considering the spatial distribution and size of the reconstituted zones?

Response:

Thanks for your comments. The research results show that the distribution of main roads and rivers is closely related, and the reconstructed area with high grade not only maintains a certain distance from the city, but also has good contact opportunities with the outside world, and the villagers are convenient to travel, which indicates the feasibility of identifying the scope boundary of rural settlement reconstruction with the accessibility measurement method to a certain extent.

2. Line 530: Please explain the meaning of the statistic “85.52%” and provide clarification on its significance.

Response:

Thanks for your comments. 85.52 represents the proportion of the area of rural reconstruction zoning to the total area of rural settlements in the study area. As shown in Figure 1, the distribution of some rural settlements in the study area is relatively scattered, and the area of the study area is relatively large, so only the reconstruction direction of most settlements with relatively concentrated distribution is determined.

**Minor:**

1. Line 108-109: All numbers above 1000 should include thousands separators, such as “1,000”.

Response:

Thanks for your comments. It has been modified according to your suggestion.

2. Line 123: Replace “Gansu Province” with “Hexi Corridor”.

Response:

Thanks for your comments. It has been modified according to your suggestion.

3. Line 145: Add “in China” after “Statistics”.

Response:

Thanks for your comments. It has been modified according to your suggestion.

4. Line 156: The “Ecosystem services value equivalent factor” column in Table 2 should be placed in a separate table between line 219 and line 220.

Response:

Thanks for your comments. It has been modified according to your suggestion.

5. Line 160 and Line 258: Replace “NNI” with the correct term.

Response:

Thanks for your comments. It has been replaced “ANN”.

6. Line 262: Consider separating the paragraph starting with “The trend surface analysis…” into separate paragraphs.

Response:

Thanks for your comments. It has been modified according to your suggestion.

7. Line 291-298: Merge the paragraph starting with “We detected two density…” with the previous paragraph.

Response:

Thanks for your comments. It has been modified according to your suggestion.

8. Line 313-322: Merge the paragraph starting with “Rural settlement cold spots…” with the previous paragraph.

Response:

Thanks for your comments. It has been modified according to your suggestion.

9. Line 349: There is an error in the format “3-km × 3-km”. Please correct it.

Response:

Thanks for your comments. It has been modified to be correct.

10. Line 392: The format “0.68·km^-2^ to 1.41·km^-2^” is incorrect. Please revise it.

Response:

Thanks for your comments. It has been modified to be correct.

11. Line 439: Replace “(” with “,”.

Response:

Thanks for your comments. It has been modified according to your suggestion.

12. Line 454- 460: Consider separating the segments of “Central village building…” to “efficient agriculture” into separate paragraphs.

Response:

Thanks for your comments. It has been modified according to your suggestion.

13. Line 461-470: Consider separating the segments of “The internally coordinated…” to “residents” into separate paragraphs.

Response:

Thanks for your comments. It has been modified according to your suggestion.

14. Line 470-481: Consider separating the segments of “With the purpose…” to “rural revitalization and development” into separate paragraphs.

Response:

Thanks for your comments. It has been modified according to your suggestion.

15. Line 538: Replace “.” with “;” to improve clarity.

Response:

Thanks for your comments. It has been modified according to your suggestion.
